# Editorial: T cell specificity and cross-reactivity – implications in physiology and pathology

**DOI:** 10.3389/fimmu.2024.1385415

**Published:** 2024-02-28

**Authors:** Daniela Latorre, Silvia Monticelli, Tomasz P. Wypych, Dominik Aschenbrenner, Samuele Notarbartolo

**Affiliations:** ^1^ Institute of Microbiology, ETH Zurich, Zurich, Switzerland; ^2^ Institute for Research in Biomedicine, Università della Svizzera italiana (USI), Bellinzona, Switzerland; ^3^ Nencki Institute of Experimental Biology, Polish Academy of Sciences, Warsaw, Poland; ^4^ Immunology Disease Area, Novartis BioMedical Research, Basel, Switzerland; ^5^ Infectious Diseases Unit, Fondazione IRCCS Ca’ Granda Ospedale Maggiore Policlinico, Milan, Italy

**Keywords:** cross-reactive T cells, T cell cross-reactivity, T cell receptor (TCR), molecular mimicry, T cell repertoire, autoimmune diseases, heterologous immunity and protection, evolution of T cell immunity

T cells are pivotal in orchestrating adaptive immune responses against a myriad of threats. Their ability to recognize antigens presented by specialized antigen-presenting cells (APCs), discern between self and non-self, and regulate and execute tailored immune responses lies at the heart of our immunological defenses. At the forefront of T cell function is the T cell receptor (TCR), a complex molecular machinery tasked with the daunting challenge of discriminating between an extensive array of antigens. Conventional CD8^+^ and CD4^+^ T cells recognize antigens exposed by APCs in the form of short peptides loaded onto major histocompatibility complex (MHC) class I and class II molecules, through their TCR (1). This interaction is key for T cell immune function as it triggers a signaling cascade that results in T cell activation, differentiation, and effector response.

The specificity of T cell-mediated immune responses is driven by the diversification of TCRs. TCRs are heterodimers of alpha and beta chains (αβTCR), which are encoded by genes on human chromosome 14 and chromosome 7, respectively. Both loci contain variable (V), joining (J), and constant (C) gene fragments, while only the TCR β locus includes two diversity (D) gene fragments between V and J. TCR diversity is determined at three different levels: the recombination of the V(D)J gene fragments, the generation of random junctional sequences by the terminal deoxynucleotidyl transferase enzyme, and the pairing of the productive alpha and beta chains. The theoretical repertoire of human T cells is enormous [possibly 10^19^-10^20^ (2, 3)], with the sole V(D)J recombination contributing with more than 5 × 10^6^ possible combinations of TCR gene fragments, assuming these are all equally possible (**Table 1**). However, 10^8^-10^10^ is the estimated number of unique TCRs surviving clonal (positive and negative) selection in the thymus and forming the mature T cell repertoire (5–7). These numbers are several orders of magnitude lower than the possible array of peptides that can be generated and accommodated into an MHC molecule (8). Considering the disparity between the number of possible foreign antigens and TCRs, the “one-clonotype–one-specificity” paradigm would result in a breach of the adaptive immune barrier.

**Table 1:** TCR diversity based on V(D)J recombination and αβ chains pairing.

Gene name	Number of functional gene segments
*TRAV*	45
*TRAJ*	50
*TRAC*	1
Possible TCRα *VJC* triplets	2,250
*TRBV*	47
*TRBD*	2
*TRBJ*	13
*TRBC*	2
Possible TCRβ *VDJC* quadruplets	2,444
Total possible TCRαβ pairs	5,499,000

Number of unique human TCRs that can be generated by the process of V(D)J recombination, without considering the junctional-derived diversity, and assuming that all the combinations of gene segments are equally possible. The indicated number of functional gene segments, based on data from the International Immunogenetics Information System (IMGT, https://www.imgt.org/IMGTrepertoire/), does not include pseudogenes and open-reading frames (ORFs). Recently published data indicate that inter-individual allelic variation in TCR genes may further broaden the TCR diversity reported here (4).

This deficit in TCR diversity is resolved by T cell cross-reactivity, that is the ability of a single TCR to bind multiple peptide-MHC complexes, albeit with different affinities. In addition to the theoretical need, numerous independent studies have experimentally demonstrated the existence of cross-reactive T cells [reviewed in (9, 10)].

In this Research Topic, we aim to offer an overview of established knowledge and recent advances in the field of T cell cross-reactivity, providing novel insights into the processes governing this phenomenon.

First, Acuto comprehensively analyzes the mechanisms regulating TCR activation and signaling portraying the context of T cell reactivity and cross-reactivity. He reviews the role of MHC-I and MHC-II, TCR αβ, and the uncertainties in understanding how peptide-MHC binding induces TCR signals. Next, he discusses various models of TCR activation, such as oligomerization, mechanotransduction, and allosteric activation, including recent evidence suggesting that TCR-CD3 activation may be controlled by an allosteric mechanism requiring only monomeric peptide-MHC binding. Finally, he proposes a unifying model for TCR activation.

TCR signaling is key for T cell activation in the periphery and governs T cell clonal selection in the thymus. Thymic selection shapes the mature T cell repertoire by eliminating non-reactive or strongly self-reactive T cell clones and directing the differentiation of conventional naïve and regulatory T (Treg) cells. Welsh et al. show that H2-O, an MHC-II peptide editing molecular chaperon, limits Treg cell differentiation in the thymus and CD4^+^ T cell hyperactivity in the periphery, possibly by modulating the range of peptides with different affinities presented on MHC-II during the thymic selection.

Still in the context of modulating TCR responses, Balasubramanian and Sundrud review the role of ATP-binding cassette (ABC) transporters in immune regulation, specifically focusing on MDR1. ABC transporters are increasingly recognized for their involvement in T cell development and function. MDR1, in particular, contributes to T cell antioxidant function, influencing TCR signaling, metabolic pathways, and oxidative stress responses. The authors discuss how MDR1 may be central to shaping the magnitude, type, and even the repertoire of T cells during antigen-specific responses and highlight the potential of ABC transporters as targets to improve therapeutic immune responses.

Cross-reactivity has evolved to cope with the enormous diversity of mutating pathogens. T cell cross-reactivity facilitates polyclonal immune responses to a single antigen and increases resistance to escape mutants. It can also induce heterologous immunity, that is the generation of memory to a pathogen different from the one against which the immune response has been originally raised. These concepts have become evident in the recent SARS-CoV-2 infection pandemics (11). Westphal et al. bring new evidence to this topic, demonstrating that CD4^+^ T cells responding to the nonstructural protein 12 (NSP12) of SARS-CoV-2 can be found both in COVID-19 patients and seronegative individuals and cross-react with the homologous protein of common-cold coronaviruses. Interestingly, they observed that NSP12 immunodominant epitopes are recognized by CD4^+^ T cells with different frequencies in COVID-19 patients and seronegative individuals and that the frequency of the response does not necessarily correlate with the NSP12 sequence conservation in different coronavirus species. These data suggest that epitope similarity is only one of the drivers of T cell cross-reactivity.

A deeper understanding of the principles underlying T cell cross-reactivity may also have implications for therapeutic applications. For instance, Bodas-Pinedo et al. explore the cross-reactivity between bacteria and viruses as a tool for designing better vaccines. Using an *in-silico* approach, they analyzed shared peptidome spaces and cross-reactive T cells between a selected bacterial consortium and the Influenza A virus and identified cross-reactivity patterns between bacterial and viral epitopes that might be harnessed to design better vaccines against flu.

Besides, using data from peptide:MHC-I and pMHC:TCR structures, Papadaki et al. identified residues important for MHC-I binding to peptides and TCR. Then, they developed a computational platform to design synthetic HLA molecules that could be used as screening tools to evaluate peptide-centric interactions with TCRs, such as for the development of improved chimeric antigen receptors (CARs).

Nonetheless, T cell cross-reactivity is a double-edged sword and can have both positive and negative consequences. Gouttefangeas et al. describe the dilemma faced by T cells that need to achieve both optimal target specificity and complete coverage of the complex spectrum of foreign antigens while avoiding reactivity to self-derived peptides. They provide an overview of the basic mechanisms underlying T cell cross-reactivity and comprehensively review its main detrimental consequence, namely the recognition of self-antigens causing autoimmunity. They also highlight a less frequently appreciated positive aspect of cross-reactivity, namely enabling T cells to recognize tumor-associated antigens.

On the same line, Thomas and Olsson focus on the role of molecular mimicry and cross-reactive T cells in the pathogenesis of multiple sclerosis (MS). They delve into the current knowledge of the intricate autoantigen repertoire targeted by autoreactive T cells in MS, detailing the evidence of cross-reactivity between antigens derived from Epstein-Barr virus (but also other microbes) and autoantigens in the host’s central nervous system (CNS). These data highlight the urgent need for further research to fully understand the role of foreign antigens in the development and progression of CNS demyelinating diseases.

Finally, Carbone et al. investigate immune-related adverse events (irAEs) in oncologic patients undergoing immunotherapy, focusing on cases of vitiligo onset in melanoma patients receiving therapeutic anti-PD-1. They show that T cells in patients with spontaneous and immunotherapy-associated vitiligo had a different immune profile, suggesting a different etiopathology of the two autoimmune clinical manifestations. Moreover, using TCR-sequencing, they find shared T cell clones in vitiligo skin lesions and metastatic (but not primary) melanoma biopsies, suggesting that an immune response against metastatic cells may trigger vitiligo development. Considering that irAEs are the main cause of immunotherapy discontinuation, increasing our understanding of the cross-reactivity of T cells to tumor-associated and self-antigens will be instrumental in developing innovative immunotherapies with limited irAEs.

In conclusion, despite all the acquired knowledge, a lot remains to be learned about cross-reactive T cells, such as phenotype and function in health and disease, TCR repertoires, and target antigens. A deeper understanding of the processes and principles associated with T cell activation, specificity, and cross-reactivity will have relevant implications in the prevention and treatment of autoimmune diseases, the development of vaccines, the optimization of engineered TCRs targeting tumor antigens, and the advancement of innovative approaches for precision medicine.

## Author contributions

SN: Conceptualization, Writing – original draft, Writing – review & editing. DL: Writing – review & editing. SM: Writing – review & editing. TW: Writing – review & editing. DA: Writing – review & editing.
